# Patent Application Trends of Induced Pluripotent Stem Cell Technologies in the United States, Japanese, and European Applications

**DOI:** 10.1089/biores.2018.0028

**Published:** 2019-03-19

**Authors:** Yasushi Morita, Hanayuki Okura, Akifumi Matsuyama

**Affiliations:** ^1^ Department of Regenerative Medicine Support Promotion Facility, Center for Research Promotion and Support, Fujita Health University, Osaka, Japan.; ^2^ Department of Regenerative Medicine and Stem Cell Biology, School of Medicine, Fujita Health University, Toyoake, Japan.

**Keywords:** disease-specific cells, European applications, induced pluripotent stem cells, Japanese application, patent application trend, U.S. application

## Abstract

Patent application trends were investigated for induced pluripotent stem cell (iPSC) technologies, particularly disease-specific cell technologies related to iPSCs, in the U.S., Japanese, and European applications during 2017. The number of patent applications for iPSC technologies was 1516 in the United States, 895 in Japan, and 420 in Europe, with 5% of applications for disease-specific cell technologies. In contrast, the percentages of patent applications for iPSC preparation and differentiation technologies were 17% and 23%, respectively. Patent applications for disease-specific cell technologies were classified into four technical fields and 14 disorder groups. In the technical fields, patent applications for genetically engineered cell technologies were prominent, accounting for 63%, 50%, and 65% of the U.S., Japanese, and European applications for 11, 8, and 7 disorder groups, respectively. In the disorder groups, the percentages of patent applications for neurological disorders were 40%, 32%, and 40% of the U.S., Japanese, and European applications, respectively, which were filed in four technical fields in the U.S. and Japanese applications. The U.S. patent applications for disease-specific cell technologies were filed by applicants in the United States, Japan, France, Belgium, Italy, Korea, and Canada; however, patent applications filed by those in Belgium, Italy, and Canada were not found in the Japanese and European applications. The percentages of patent applications filed by the U.S. applicants were 72%, 55%, and 65% of the U.S., Japanese, and European applications, respectively. Most patent applications filed by the U.S. applicants were in the field of genetically engineered cells for 11 disorder groups, which mostly included neurological and blood disorders. Japanese applicants mainly filed patent applications for drug screening technologies; subjects included five disorder groups, particularly neurological and bone/articular disorders. French applicants filed patent applications for neurological disorders in the field of genetically engineered cells and drug screening technologies. Korean applicants filed patent applications for patient-derived cell technologies for neurological, metabolic, and chromosomal/genetic disorders. In conclusion, more than half of patent applications were for genetically engineered cells for 11 disorders, most of which were filed by U.S. applicants.

## Introduction

Since the first report^1^ of induced pluripotent stem cells (iPSCs) in 2006, these cells have been used to develop regenerative medicines and therapies. Indeed, the first clinical trial study in humans for age-related macular degeneration using iPSC-derived retinal pigment epithelial cells was conducted in 2014, with the results published in 2017.^2^ In addition to their utility as stem cell sources for therapies, iPSCs are useful for drug development^3^ and disease modeling.^4,5^ Recently, Negoro et al.^6^ reported on research article trends in iPSCs focused on drug development and pathological studies of disease using the PubMed database.

Freedom to operate (FTO) investigations are essential for research, clinical trials, and commercialization of iPSCs. Few studies have examined patent trends for stem cells^7^ and iPSCs^8^ through 2013; patent trends in iPSC technologies and patent trends in disease-specific cell technologies related to iPSCs have not been found.

In this study, published patent applications filed with the U.S. Patent and Trademark Office (USPTO), Japan Patent Office, and European Patent Office describing iPSC in claims were searched, and patent application trends in iPSC technologies, particularly disease-specific cell technologies related to iPSCs, were investigated.

## Materials and Methods

Keyword searches were conducted for claims of published patent applications filed with the U.S., Japanese, and European applications published from January 1, 2006, to December 31, 2017. The European applications were searched for only the publications described in English. The applications included ordinary, divisional, continuation, and continuation-in-part applications.

1. Database: USPTO Patent Application Full-Text and Image Database (AppFT), J-PlatPat, and Patentscope.
2. Query: “induced pluripotent stem cell” OR “iPS cell” OR “iPSC” in claims of published patent applications.

The published patent publications were manually reviewed and those for iPSC preparation technologies, differentiation technologies of iPSCs, and disease-specific cell technologies were extracted. Furthermore, the U.S., Japanese, and European applications were checked for examination status using Patent Application Information Retrieval (Public PAIR), Legal Status in J-PlatPat, and Global Dossier in Espacenet, respectively.

## Results

### Patent application trends in iPSC technologies

**FIG. 1.**
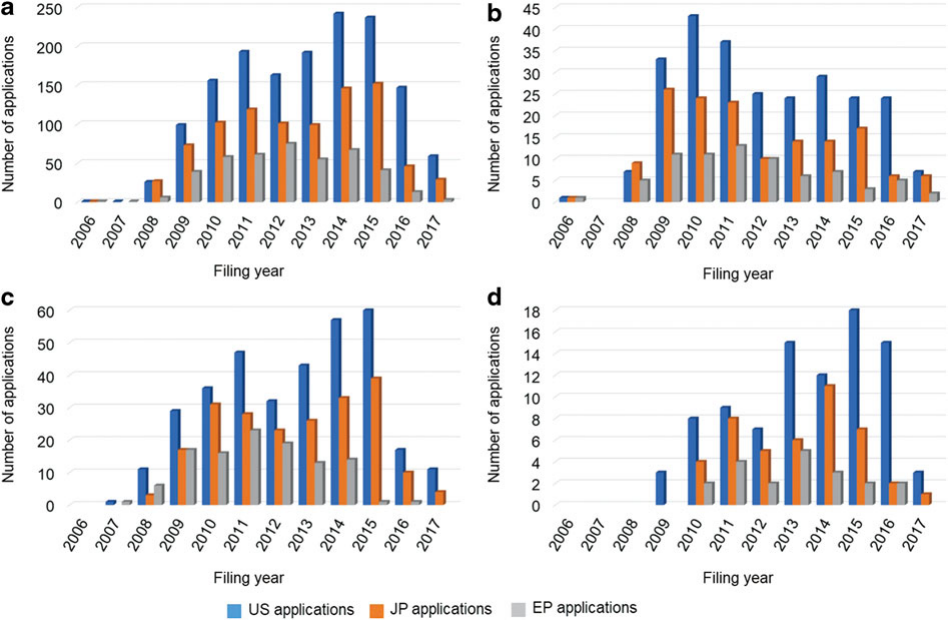
Trends in patent applications for total iPSC technologies **(a)**, iPSC preparation technologies **(b)**, differentiation technologies **(c)**, and disease-specific cell technologies **(d)** by filing year. EP, European; iPSC, induced pluripotent stem cell; JP, Japanese; U.S., United States.

Since 2006 when Kyoto University filed its first patent application for iPSCs,^9^ the number of patent applications for iPSC technologies increased dramatically through 2011 and then showed a slow increase (Fig. 1a). The notable decreases in the number of patent publications in 2016 and 2017 may be related to the time lag until publication. In general, a patent application is published 1.5 years after the filing date or priority date. Furthermore, a patent application filed under the Patent Cooperation Treaty has to enter the national phase in the United States, Japan, and Europe within 30 months from the priority date.

Patent applications for iPSC technologies were filed by applicants in the United States, Japan, Austria, Belgium, Switzerland, Germany, Denmark, Spain, Finland, France, the United Kingdom, Hungary, Italy, Netherlands, Norway, Sweden, Portugal, Bangladesh, China, Hong Kong, Israel, India, Republic of Korea, Singapore, Turkey, Taiwan, Australia, and Canada. The number of patent applications by applicant nationality is shown in Table 1. For U.S. applications, the total number of patent applications was 1516, with 61% filed by U.S. applicants, 17% filed by Japanese applicants, 9% filed by European applicants from 13 countries, 11% filed by Asian applicants from 9 countries except Japan, and 2% filed by Australian and Canadian applicants. For Japanese applications, the total number of patent applications was 895, which is roughly 60% of the total number of U.S. applications. Of the 895 Japanese applications, 53% were filed by Japanese applicants, 32% filed by U.S. applicants, 9% filed by European applicants from 11 countries, 5% filed by Asian applicants from 6 countries, and 1% filed by Australian and Canadian applicants. For European applications, the total number of patent applications was 420, which is roughly 28% of the total number of U.S. applications. Of these 420 European applications, 37% were filed by Japanese applicants, 32% filed by U.S. applicants, 18% filed by European applicants from 11 countries, and 12% filed by Asian applicants from 6 countries.

**Table 1.** Number of Patent Applications for Induced Pluripotent Stem Cell Technologies by Each Applicant's Nationality

	Number of applications																											
	US	JP	AT	BE	CH	DE	DK	ES	FI	FR	GB	HU	IT	NL	NO	SE	PT	BD	CN	HK	IL	IN	KR	SG	TR	TW	AU	CA
USPTO	921	253	1	8	5	28	2	5	8	25	39	1	4	2	1	9	1	1	29	2	21	1	58	30	1	20	4	36
JPO	287	469	2	1	11	12	1	1	3	19	19			2		8			12		11		12	8	1	4	2	10
EPO	135	158	3	1	6	25	1	2	4	12	15		3			3			15		2	1	21	7		3		3

AT, Austria; AU, Australia; BD, Bangladesh; BE, Belgium; CA, Canada; CH, Switzerland; CN, China; DE, Germany; DK, Denmark; EPO, European Patent Office; ES, Spain; FI, Finland; FR, France; GB, United Kingdom; HK, Hong Kong; HU, Hungary; IL, Israel; IN, India; IT, Italy; JP, Japan; JPO, Japan Patent Office; KR, Republic of Korea; NL, Netherlands; NO, Norway; PT, Portugal; SE, Sweden; SG, Singapore; TR, Turkey; TW, Taiwan; US, United States; USPTO, U.S. Patent and Trademark Office.

Changes in the number of patent applications for iPSC preparation technologies, differentiation technologies, and disease-specific cell technologies by filing year are shown in Figure 1b–d, respectively. The number of patent applications for iPSC preparation technologies increased rapidly until 2010 and then decreased gradually; the annual trend of the number of patent applications for differentiation technologies has increased rapidly until 2011 and then increased gradually until 2015. Although the number of patent applications for disease-specific cell technologies was small compared with that for iPSC preparation and differentiation technologies, the number of patent applications filed from 2009 to 2015 has gradually increased. For each technology, 17% of patent applications were for iPSC preparation technologies, 23% for differentiation technologies of iPSCs, and 5% for disease-specific cell technologies.

### Patent application trends in iPSC preparation technologies

Since Yamanaka at Kyoto University filed a patent application for four nuclear reprogramming factors, Oct-3/4, Klf4, c-Myc, and Sox2, for generating iPSCs in 2006,^9^ many patent applications for iPSC preparation methods have been filed. In this study, iPSC preparation methods were classified into the following six categories: (1) a method using one or more reprogramming factors selected from Oct, Klf, Myc, Sox, Nanog, and Lin 28 genes (referred to as “RFs”), (2) a method using a combination of reprogramming factors and other transcription factors or chemical compounds and such as reprogramming enhancers (“RFs + others”), (3) a method using RNAs selected from synthesized modified RNAs and microRNAs (miRNAs), (4) a method using episomal vectors loaded with reprogramming factors, (5) a method using Sendai virus vectors loaded with reprogramming factors, and (6) miscellaneous.

**FIG. 2.**
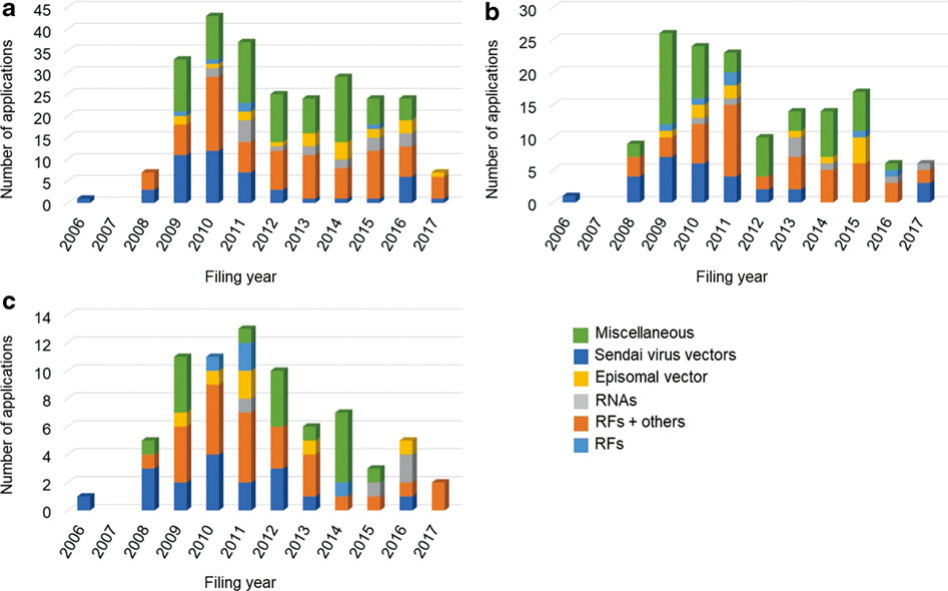
Trends in patent applications for each iPSC preparation method by filing year. **(a)** U.S. applications, **(b)** JP applications, and **(c)** EP applications.

Figure 2 shows the trends in patent applications for each iPSC preparation method by filing year. Table 2 shows the total number of applications for each method by applicant nationality. Approximately 30% of the patent applications were for iPSC preparation methods using “RFs + others,” followed by the methods using RFs, RNAs, episomal vectors, and Sendai virus vectors. The number of patent applications for the method using RFs peaked in 2010 and then decreased. The number of patent applications for the method using “RFs + others” also peaked in 2010 and then decreased moderately compared with that for the method using RFs. The annual number of patent applications for the iPSC preparation method using RNAs, episomal vectors, and Sendai virus vectors was small. The patent applications for the method using RNAs and episomal vectors were filed from 2010 to 2015 by mainly U.S. applicants, and the patent applications for the method using Sendai virus vectors were filed by Japanese applicants between 2009 and 2011.

**Table 2.** Number of Patent Applications for Each Induced Pluripotent Stem Cell Preparation Method by Applicant's Nationality

	Number of patent applications								Number of granted patents							
	US	JP	GB	FR	DE	CN	KR	Other	US	JP	GB	FR	DE	CN	KR	Other
USPTO																
RFs	24	12	2	2	3		1	3	2	7	1	1	1			2
RFs + others	44	16	1	4	1	5	4	9	20	11	1	2		2	1	3
RNAs	16	1			1				9	1						
Episomal vector	16	2						1	9	1						1
Sendai virus vectors		5								5						
Miscellaneous	53	11	1	1	1	5	7	2	21	4				2	3	
JPO																
RFs	6	18		2	1			2		12						
RFs + others	15	21	1	2		2	2	3	7	13	1	1				
RNAs	6	2								2						
Episomal vector	8	3							4							
Sendai virus vectors		6								4						
Miscellaneous	20	19		3	2	1	3	2	4	8				1	1	1
EPO																
RFs	4	6	1	1	4	1			2	5	1	1	1			
RFs + others	11	9				3	1	2	7	5				2	1	1
RNAs	3	1														
Episomal vector	5	1							4							
Sendai virus vectors		4								3						
Miscellaneous	6	2		1	1	2	5		5	1		1		2	2	

Other: CH, ES, IT, IL, SG, TW, and CA in USPTO; CH, ES, IL, SG, and CA in JPO; SG and TW in EPO.

For the iPSC preparation methods using “RFs + others,” Kyoto University has 10 granted patents in the United States, 11 in Japan, 5 in Europe for the combination of RFs and histone deacetylase inhibitors,^10^ miRNAs,^11^ p53 function inhibitors,^12,13^ GLIS family member jointly with the National Institute of Advanced Industrial Science and Technology and Japan Biological Informatics Consortium,^14,15^ cyclin D family member,^16^ p38 function inhibitors,^17^ and AKT family member.^18^ The Scripps Research Institute has eight granted patents in the United States, five in Japan, and three in Europe for the combination of RFs and GSK-3 inhibitors,^19^ MEK inhibitors,^20^ ALK5 inhibitors,^21^ and TGFβ receptor/ALK5 inhibitors.^22^

The University of Pennsylvania has five granted patents in the United States for iPSC preparation methods using synthesized modified messenger RNAs^23^ and miRNAs,^24^ and Osaka University has one granted patent each in the United States and Japan for miRNA methods.^25^

Cellular Dynamics International, Inc. has nine granted patents in the United States and three in Japan and Europe for iPSC preparation methods using episomal vectors.^26–31^ For iPSC preparation methods using Sendai virus vectors, Denavec has been granted patents in the United States, Japan, and Europe, including joint patents with Keio University,^32,33^ while the National Institute of Advanced Industrial Science and Technology has been granted patents in the United States, Japan, and Europe, including a joint patent with the Japan Biological Informatics Consortium.^34–36^

### Patent application trends in iPSC differentiation technologies

Cells differentiated from iPSCs were classified into seven groups: neuronal, cardiac, pancreatic, hepatic, hematopoietic, ectoderm/mesoderm/endoderm, and miscellaneous cells. In this search, the neuronal cell group included neural, neural stem, neural precursor, neural crest, dopaminergic neuronal, oligodendroglial precursor, orexin neuron, and motor neuron cells; cardiac cells included cardiogenic progenitor cells and cardiomyocytes; pancreatic cells included pancreatic progenitor, insulin secreting, GLUT2-expressing, and pancreatic hormone-producing cells; hepatic cells included hepatocyte, hepatic stem, and hepatic progenitor cells; hematopoietic cells included hematopoietic stem and hematopoietic precursor cells; mesoderm/ectoderm/endoderm cells included mesodermal, mesodermal progenitor, intermediate mesoderm, ectodermal, ectodermal progenitor, endodermal, endodermal progenitor, definitive endoderm, and anterior foregut endoderm cells. The miscellaneous group contained 30 differentiated cells, including mast, eosinophil, dendritic, and T and natural killer cells, megakaryocytes and platelets, blood, erythroid, erythropoietin-producing, skeletal muscle, smooth muscle, myeloid, osteoblast, chondrogenic, corneal epithelial, corneal endothelial, retinal, retinal ganglion, retinal stem, retinal pigment epithelial, epiblast-like, endothelial, mesenchymal stem, and somatic cells.

**FIG. 3.**
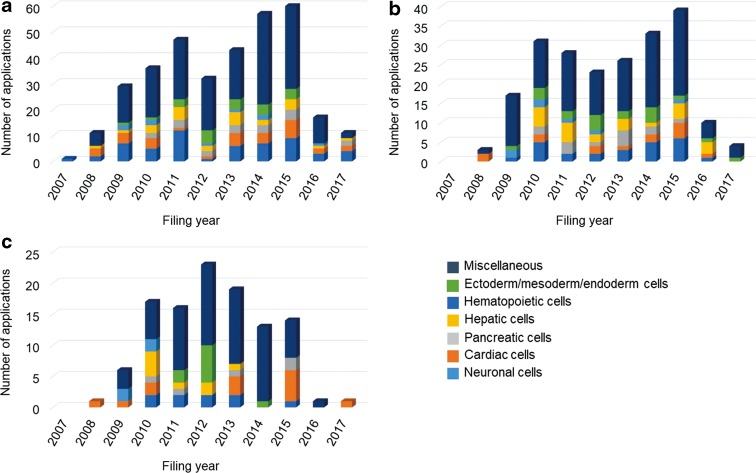
Trends in patent applications for each cell differentiated from iPSCs by filing year. **(a)** U.S. applications, **(b)** JP applications, and **(c)** EP applications.

Figure 3 shows the trends in patent applications for each cell differentiated from iPSC by filing year. Table 3 shows the total number of applications for each cell, differentiated from iPSCs by applicant nationality.

**Table 3.** Number of Patent Applications for Each Cell Differentiated from Induced Pluripotent Stem Cell by Applicant's Nationality

	Number of patent applications								Number of granted patents							
	US	JP	GB	FR	DE	CN	KR	Other	US	JP	GB	FR	DE	CN	KR	Other
USPTO																
Neuronal cells	40	4	2	1			5	5	16	1					3	2
Cardiac cells	21	6				2		4	9	2				1		
Pancreatic cells	12	3	1				1	2	6	2	1				1	1
Hepatic cells	7	6	1		1	2	1	7	3	1	1					4
Hematopoietic cells	7	1				1			2							
Ectoderm/mesoderm/endoderm cells	9	4	2	3			2	2	6	2	1				1	1
Miscellaneous	88	46	10	4	2	2	8	19	38	18	4	1	2	1	5	7
JPO																
Neuronal cells	12	6	3	2			1	2	3	4		1			1	1
Cardiac cells	1	7			1	3		1	1	3						
Pancreatic cells	3	7	1	1				5		3						1
Hepatic cells	2	14	1			2		4	1	6	1					3
Hematopoietic cells	3	4							2	4						
Ectoderm/mesoderm/endoderm cells	4	8	1	1				5	1	3						3
Miscellaneous	25	73	3	3			1	8	8	36						3
EPO																
Neuronal cells	3	4					2		3	2					1	
Cardiac cells	4	6				1		2	2	3						1
Pancreatic cells		3	1					1		2	1					
Hepatic cells		2	1		1	1	1	2			1					2
Hematopoietic cells	2	2							2	1						
Ectoderm/mesoderm/endoderm cells	1	4		2				2		3						
Miscellaneous	12	30	6		4		4	7	9	13	4		2		1	4

Other: BE, DK, ES, FI, IT, SE, NO, BD, IL, SG, TW, AU, and CA in USPTO; AT, CH, DK, SE, IL, SG, TW, and CA in JPO; AT, CH, DK, ES, ES, FI, IT, SE, IL, SG, and CA in EPO.

For U.S. applications, 17% were for neuronal cells, followed by cardiac, hepatic, ectoderm/mesoderm/endoderm, pancreatic, and hematopoietic cells. In the miscellaneous group, the number of applications decreased for megakaryocytes and platelets, T cells, osteoblasts and chondrocytes, retinal pigment epithelial cells, and mesenchymal stem cells in order.

For Japanese applications, the percentage of patent applications decreased in the order of neuronal (12%), hepatic (11%), ectoderm/mesoderm/endoderm, cardiac, pancreatic, and hematopoietic cells. In the miscellaneous group, as in U.S. applications, in descending order, the number of applications was megakaryocytes and platelets, T cells, osteoblasts and chondrocytes, and retinal pigment epithelial cells.

For European applications, the percentage of patent applications decreased in the order of cardiac (21%), neuronal, ectoderm/mesoderm/endoderm, hepatic, pancreatic, and hematopoietic cells. The applications for megakaryocytes and platelets, T cells, and osteoblasts and chondrocytes were also noticeable in the miscellaneous group.

Kyoto University, Cellular Dynamics International, Inc., the University of Tokyo, and Wisconsin Alumni Research Foundation are the leading patentees of these technologies. Kyoto University has 11 granted patents in the United States, 13 in Japan, and 7 in Europe for neural precursor cells,^37^ cardiogenic progenitor cells, and/or cardiomyocytes,^38^ insulin producing cells jointly with Kumamoto University,^39^ intermediate mesoderm cells,^40,41^ dendritic cells,^42^ eosinophils,^43^ skeletal muscle or skeletal progenitor cells,^44,45^ germ cell-like cells,^46,47^ erythropoietin-producing cells,^48^ mast cells,^49^ and somatic cells.^50,51^ Cellular Dynamics International, Inc. has 11 granted patents in the United States, 7 in Japan, and 5 in Europe for neural cells,^52,53^ cardiomyocytes,^54^ hepatocytes,^55^ hematopoietic precursor cells,^56,57^ endothelial cells,^58^ mast cells,^59^ and keratinocytes.^60^ The University of Tokyo has 4 granted patents in the United States, 10 in Japan, and 3 in Europe for orexin neurons,^61,62^ hepatocytes or pancreatic cells,^63^ megakaryocytes and/or paltelets,^64–66^ T cells,^67,68^ blood cells,^69^ and erythroid cells.^70^ The University of Tokyo has been granted a patent for megakaryocytes and/or platelets jointly with Nissan Chemicals Corporation.^71^ Wisconsin Alumni Research Foundation has nine granted patents in the United States and one in Europe for neural stem cells,^72^ oligodendroglial precursor cells,^73^ cardiomyocytes,^74^ pancreatic lineage cells,^75,76^ hepatocytes,^77^ blood/brain barrier endothelial cells,^78^ myeloid lineage cells,^79^ and mesenchymal stem cells.^80^

### Patent application trends in disease-specific cell technologies

Disease-specific cell technologies were categorized into four technical fields: genetically engineered cell technologies, patient-derived cell technologies, drug-screening technologies, and diagnosis technologies. Furthermore, disorders in disease-specific cell technologies were categorized into 14 groups with reference to the classifications by the Japan Intractable Disease Information Center (website in Japanese) as follows: neurological, blood, metabolic, chromosomal/genetic, immune, cardiovascular, ophthalmological, skin, digestive, endocrine, bone/cartilage, and renal/urological disorders, cancer, and infection. In this search, neurological disorders included Alzheimer's, Huntington's, and Parkinson's disease, Duchenne muscular, muscular, and Becker muscular dystrophy, spinal cord injury, myotonic dystrophy-1, spinal muscular atrophy, amyotrophic lateral sclerosis, intellectual disability, autism, autism spectrum disorder, Rett syndrome, frontotemporal lobar degeneration, and Charcot-Marie-Tooth disease. Blood disorders included hemoglobinopathy, hemophilia A, hemophilia B, thrombocytopenia, Fanconi anemia, and acute myeloid leukemia. Metabolic disorders included lysosomal storage and Fabry diseases. Chromosomal/genetic disorders included Patau, Edwards, Down, and cardiofaciocutaneous syndromes. Immune disorders included severe combined immunodeficiency, autoimmune disease, human leukocyte antigen (HLA)-related disease, graft-versus-host disease, and chronic infantile neurologic cutaneous articular syndrome. Cardiovascular disorders included cardiomyopathy and dilated cardiomyopathy. Infection included human immunodeficiency virus infection. Ophthalmological disorders included Leber's congenital amaurosis and retinitis pigmentosa. The skin disorder was epidermolysis bullosa. The digestive disorder was cystic fibrosis. The endocrine disorder was type 2 diabetes. Bone/cartilage disorders included cartilaginous hyperplasia and fibroblast growth factor receptor 3 disease. The renal/urological disorder was polycystic kidney disease.

**FIG. 4.**
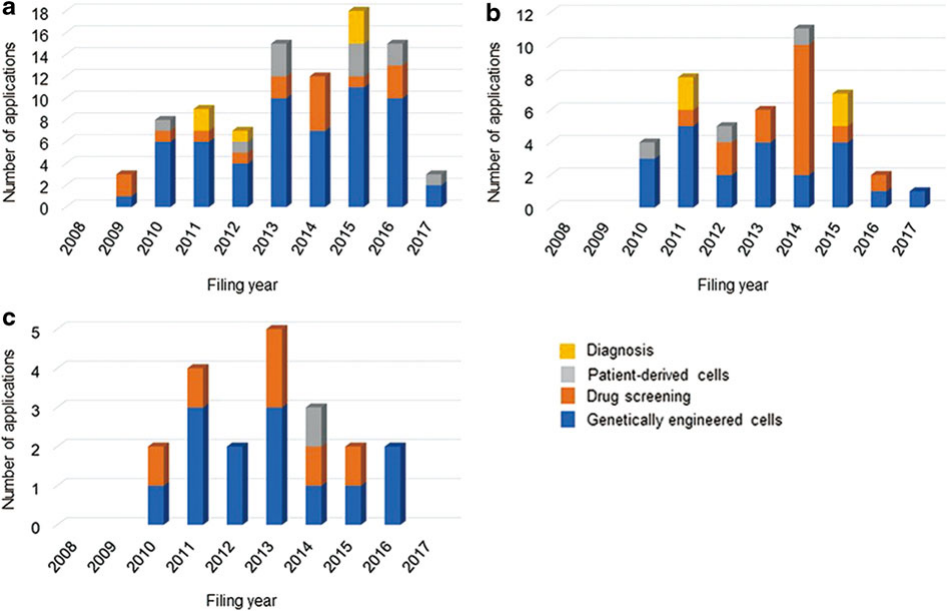
Trends in patent applications for disease-specific cell technologies for each technical field by filing year. **(a)** U.S. applications, **(b)** JP applications, and **(c)** EP applications.

**FIG. 5.**
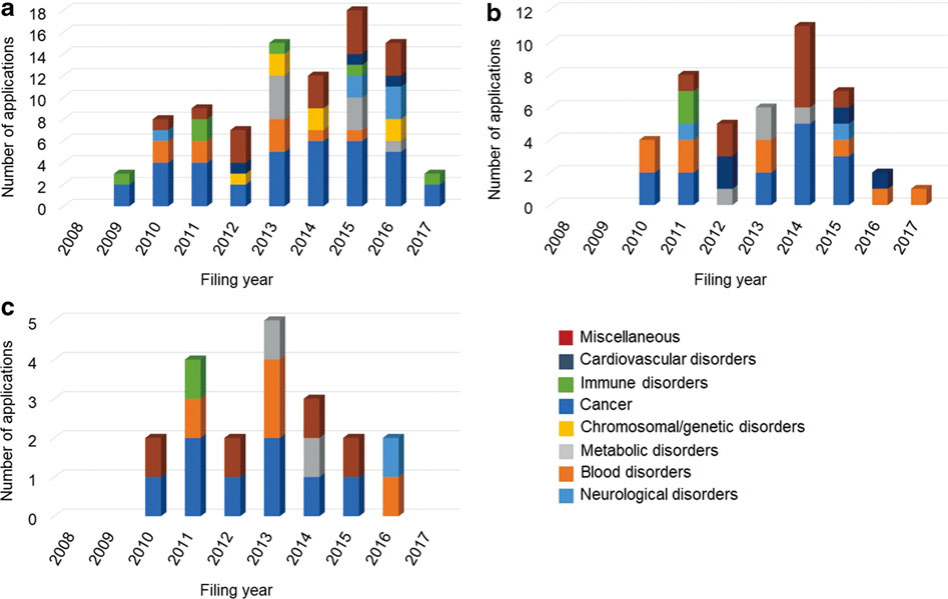
Trends in patent applications for disease-specific cell technologies for each disorder group by filing year. **(a)** U.S. applications, **(b)** JP applications, and **(c)** EP applications.

Figures 4 and 5 show the trends in patent applications for disease-specific cell technologies for each technical field and for each disorder group by filing year. Tables 4 and 5 show the number of patent applications for disease-specific cell technologies for each technical field and for each disorder group by applicant nationality.

**Table 4.** Number of Patent Applications for Disease-Specific Cell Technologies for Each Technical Field by Applicant's Nationality

Technical fields	Number of patent applications							Number of granted patents						
	US	JP	FR	BE	IT	KR	CA	US	JP	FR	BE	IT	KR	CA
USPTO														
Genetically engineered cell	49	1	1	1	1		4	15		1				2
Drug screening	6	7	2			1		4	1					
Patient-derived cells	6	1				4		1						
Diagnosis	4	2						2	1					
JPO														
Genetically engineered cell	20		2					7		1				
Drug screening	3	10	2						3					
Patient-derived cells		2				1			1					
Diagnosis	1	3							2					
EPO														
Genetically engineered cell	11		1			1		7		1				
Drug screening	2	4							2					
Patient-derived cells						1								
Diagnosis														

**Table 5.** Number of Patent Applications for Disease-Specific Cell Technologies for Each Disorder Group by Applicant's Nationality

Technical fields	Number of patent applications							Number of granted patents						
	US	JP	FR	BE	IT	KR	CA	US	JP	FR	BE	IT	KR	CA
USPTO														
Neurological disorders	25	5	3	1		1	1	7	1	1				
Blood disorders	8		1					3						
Metabolic disorders	4						2	1						1
Chromosomal/genetic disorders	5					2		3						
Cancer	5				1			1						
Immune disorders	4	1					1	4	1					1
Cardiovascular disorders	3							1						
Miscellaneous	11	4						2						
JPO														
Neurological disorders	5	6	3					1	2	1				
Blood disorders	9							3						
Metabolic disorders	2	1				1			1					
Cancer	1		1					1						
Immune disorders	1	1						1	1					
Cardiovascular disorders	3	1						1	1					
Miscellaneous	3	6							1					
EPO														
Neurological disorders	3	3	1					1	1					
Blood disorders	3					1		2						
Metabolic disorders	1					1		1						
Cancer	1							1						
Immune disorders	1	1						1	1					
Miscellaneous	3	1						2						

The first patent applications for disease-specific cell technologies were filed in 2009 in the United States and in 2010 in Japan and Europe. The U.S. patent applications for disease-specific cell technologies were filed by applicants from the United States, Japan, France, Belgium, Italy, Korea, and Canada; however, patent applications filed by applicants in Belgium, Italy, and Canada were not found in the Japanese and European applications. The percentage of patent applications filed by U.S. applicants was 72% for U.S. applications, 55% for Japanese applications, and 65% for European applications.

**FIG. 6.**
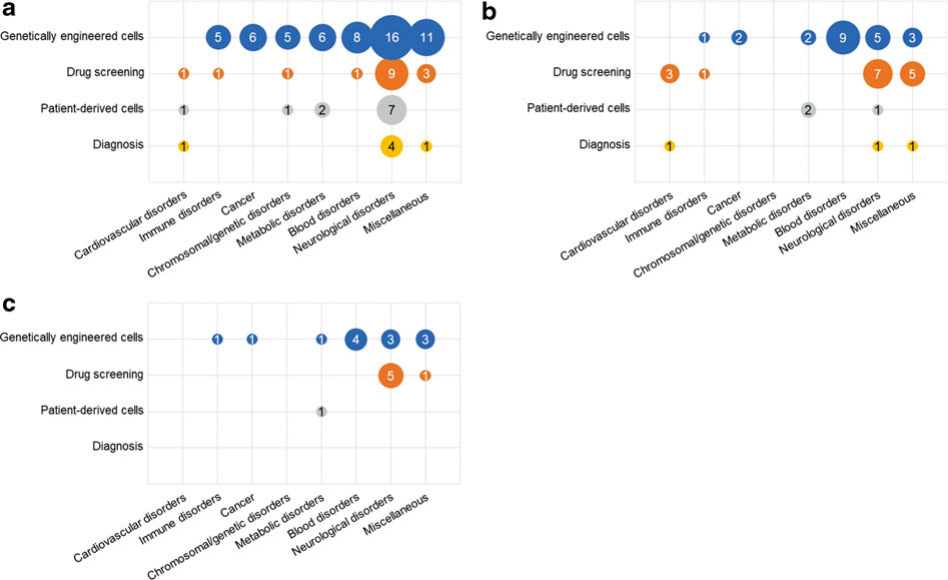
Distribution of patent applications for disorder groups in each technical field. **(a)** U.S. applications, **(b)** JP applications, and **(c)** EP applications.

For U.S. applications, the annual number of applications for genetically engineering cell technologies and for neurological disorders has been increasing; however, the trends were not found in Japanese and European applications. Figure 6 shows the distribution of patent applications for disorder groups in each technical field. In the four technical fields, the number of patent applications for genetically engineered cell technologies was highest with 57 (63%) in U.S. applications, 22 (50%) in Japanese applications, and 13 (65%) in European applications. Patent applications for genetically engineered cells were for 11 disorder groups: neurological disorders, blood disorders, metabolic disorders, chromosomal/genetic disorders, cancer, immune disorders, infection, ophthalmological disorders, skin disorders, digestive disorders, and endocrine disorders. Patent applications for drug screening technologies were for neurological, blood, chromosomal/genetic, immune, cardiovascular, and bone/articular disorders. The applications for neurological disorders were notable; the applications were filed in four technical fields in U.S. and Japanese applications and in two technical fields in European applications. The number of applications for blood disorders mainly filed in genetically engineered cell fields followed the number for neurological disorders.

For applications filed by the U.S. applicants, 75%, 83%, and 85% of U.S., Japanese, and European applications, respectively, were for genetically engineered cell technologies for the 11 disorder groups listed above. Japanese applicants mainly filed applications for drug screening technologies for neurological, blood, immune, cardiovascular, and bone/articular disorders. French applicants filed applications for neurological disorders in the field of genetically engineered cells and drug screening technologies. Korean applicants filed applications for patient-derived cell technologies for neurological, metabolic, and chromosomal/genetic disorders.

Table 6 shows representative applicants in each country and the number of patent applications. In Table 6, the number of patent applications includes joint patent applications. Sangamo BioSciences, Inc. has been granted patents for Huntington's disease,^81,82^ severe combined immunodeficiency,^83^ cancer,^84^ cystic fibrosis,^85^ and HLA-related diseases, and graft-versus-host disease, jointly with the University of Texas System.^86^ Sangamo BioSciences, Inc. and the Children's Hospital Philadelphia have been jointly granted patents for hemophilia B^87^ and metabolic disorders.^88^ The Children's Hospital Philadelphia has been granted patents for thrombocytopenia.^89^ Parkinson's Institute has been granted patents for Parkinson's disease in the field of drug screening technologies^90^ and diagnosis technologies.^91^ The Leland Stanford Junior University has been granted a patent for dilated cardiomyopathy in the field of drug screening technologies.^92^ Parkinson's Institute and the Leland Stanford Junior University have been granted a joint patent for Parkinson's disease in the field of patient-derived cell technologies.^93^ The University of Massachusetts has been granted patents for Patau, Edward, and Down syndromes.^94,95^ Kyoto University has been granted patents for amyotrophic lateral sclerosis,^96,97^ cryopyrin-associated periodic syndrome,^98^ and cardiomyopathy^99^ in the field of drug screening technologies, and for mitochondrial disease^100^ in the field of patient-derived cell technologies, as well as for polycystic kidney disease^101^ and Alzheimer's disease jointly with Riken^102^ in the field of diagnosis technologies.

**Table 6.** Representative Applicants for Disease-Specific Cell Technologies in Each Country and Number of Patent Applications

Applicants	Technical field	Number of patent applications					
		Granted			Under examination		
		USPTO	JPO	EPO	USPTO	JPO	EPO
Sangamo BioSciences, Inc.	Genetically engineered cells	9	5	5	7	5	1
Parkinson's Institute	Genetically engineered cells				2		
	Drug screening	1					
	Patient-derived cells	1			1		
	Diagnosis	2					
Stanford University	Drug screening	2				2	
	Patient-derived cells	1			2		
Children's Hospital Philadelphia	Genetically engineered cells	4	2	1	1	1	
University of Massachusetts	Genetically engineered cells	3			2		
University of Minnesota	Genetically engineered cells	1			2	1	
University of California	Genetically engineered cells				2		1
	Drug screening				1		
Kyoto University	Genetically engineered cells				1		
	Drug screening	1	3	2	5	6	3
	Patient-derived cells		1		1		
	Diagnosis	1	2		1	1	
Innovative Concepts in Drug Development	Drug screening				2	2	
Association Institut de Myologie	Genetically engineered cells	1	1	1			
Korea Advanced Institute of Science and Technology	Drug screening				1		
	Patient-derived cells				2		1
Korea Research Institute of Bioscience and Biotechnology	Patient-derived cells				1		
University Health Network	Genetically engineered cells	2					

## Discussion

iPSC technologies are categorized broadly into two categories, basic and applied technologies. Basic technologies for iPSCs include methods for preparation, differentiation, culture, separation and purification, quality control, and others. Applied technologies using iPSCs include research and development such as cell therapy, drug discovery, and disease modeling. In this report, we extracted patent applications for technologies related to iPSC preparation, iPSC differentiation methods, and disease-specific cells, and investigated patent application trends for these technologies.

In patent applications for iPSC technologies, 17% were iPSC preparation technologies, 23% were iPSC differentiation technologies, and 6% were disease-specific cell technologies. For the trend in the number of patent applications for each filing year, the number of applications for iPSC preparation decreased from 2010 and those for iPSC differentiation technologies increased until 2015. In contrast, although the total number of patent applications was low, those for disease-specific cells gradually increased. This result suggests that the research of disease-specific cells has advanced.

For disease-specific cells, iPSCs generated from patients with adenosine deaminase deficiency-related severe combined immunodeficiency, Shwachman–Bodian–Diamond syndrome, Gaucher disease type 3, Duchenne and Becker muscular dystrophy, Parkinson's disease, Huntington's disease, juvenile-onset, type 1 diabetes mellitus, Down syndrome, and Lesch–Nyhan syndrome were reported,^103^ and the first patent applications for drug screening methods for Parkinson's disease (US20100167286, now US8669048^90^) and spinal muscular atrophy^104^ were filed in 2009.

Papapertrou reported that iPSCs and the CRISPR-Cas9 system democratized stem cell modeling and genome editing, respectively.^105^ Takahashi and Yamanaka reviewed iPSC preparation mediated by transcription factors in the past 10 years and reported that the potential of iPSCs in clinical applications and disease models was strengthened by combining iPSC technology and genome engineering.^106^ Indeed, in patent applications for disease-specific cell technologies in this search, more than half were for genetically engineered cell technologies. Editas Medicine, Inc. has filed patent applications for treatment of cystic fibrosis^107^ and Leber's congenital amaurosis,^108^ using CRISPR/Cas-related methods.

To accelerate the research and development of iPSCs, iPSC banks have been constructed as a source for drug development, disease modeling, and cell therapy.^109^ If licensed iPSCs provided by the bank for research, clinical trials, and commercialization are used, caution should be taken to prevent patent infringement, as patents on iPSC technologies consist of several different technologies owned by several patent holders; therefore, it is important to consider a patent pool when using iPSC technologies. iPS Academia Japan has sublicensing rights to patent and patent applications from Kyoto University and other university and research institutes and has released a license program. It is also necessary to consider patent validities and disputes.^8,110^ For the CRISPR/Cas-9 patent, for example, the USPTO determined that there was no interference-in-fact for claims between the University of California, Berkeley and Broad Institute and maintained the patents granted to the Broad Institute.^111,112^ At the appeal trial of this case, the United States Court of Appeals for the Federal Circuit affirmed the USPTO decision.^113^

## Conclusion

In this search, more than half of the patent applications for disease-specific cell technologies were for genetically engineered cells for numerous disorders. In the future, patent applications for disease-specific cells will combine iPSC technologies and genome editing, particularly the CRISPR/Cas system. To promote the research and development of disease-specific cell-related iPSCs, FTO investigations are important, particularly for dissolving patent disputes. We hope that patent disputes surrounding iPSC technologies and genome editing will be solved and that iPSCs, principally disease-specific cells, will become a tool used in clinical applications, disease modeling, and drug development.

## Abbreviations Used

EPO: European Patent Office
FTO: Freedom to Operate
iPSC: induced pluripotent stem cell
JPO: Japan Patent Office
miRNAs: microRNAs
USPTO: U.S. Patent and Trademark Office
